# Estimating the Force of Infection for Dengue Virus Using Repeated Serosurveys, Ouagadougou, Burkina Faso

**DOI:** 10.3201/eid2701.191650

**Published:** 2021-01

**Authors:** Jacqueline K. Lim, Mabel Carabali, Tansy Edwards, Ahmed Barro, Jung-Seok Lee, Desire Dahourou, Kang Sung Lee, Teguewende Nikiema, Mee Young Shin, Emmanuel Bonnet, Therese Kagone, Losseni Kaba, Suk Namkung, Paul-André Somé, Jae Seung Yang, Valéry Ridde, In-Kyu Yoon, Neal Alexander, Yaro Seydou

**Affiliations:** International Vaccine Institute, Seoul, South Korea (J.K. Lim, M. Carabali, J.-S. Lee, K.S. Lee, M.Y. Shin, S. Namkung, J.S. Yang);; London School of Hygiene and Tropical Medicine, London, UK (J.K. Lim, T. Edwards, N. Alexander);; Centre MURAZ, Bobo-Dioulasso, Burkina Faso (D. Dahourou, T. Nikiema, T. Kagone, Y. Seydou);; McGill University, Montreal, Quebec, Canada (M. Carabali);; Action, Gouvernance, Intégration, Renforcement Program Equité, Ouagadougou, Burkina Faso (A. Barro, P.-A. Somé);; Institut de Recherché en Sciences de la Santé, Ouagadougou (D. Dahourou); I; nstitute for Research on Sustainable Development, Université de Paris, Paris, France (E. Bonnet, V. Ridde);; Centre National de Transfusion Sanguine, Ouagadougou (L. Kaba);; Coalition for Epidemic Preparedness Innovations, Washington, DC, USA (I.-K. Yoon)

**Keywords:** Aedes mosquitoes, Africa, Burkina Faso, cross reactivity, dengue, flaviviruses, force of infection, IgG, outbreaks, population seroprevalence, regression models, seroconversion, viruses

## Abstract

Because of limited data on dengue virus in Burkina Faso, we conducted 4 consecutive age-stratified longitudinal serologic surveys, ≈6 months apart, among persons 1–55 years of age, during June 2015–March 2017, which included a 2016 outbreak. The seroconversion rate before the serosurvey enrollment was estimated by binomial regression, taking age as the duration of exposure, and assuming constant force of infection (FOI) over age and calendar time. We calculated FOI between consecutive surveys and rate ratios for potentially associated characteristics based on seroconversion using the duration of intervals. Among 2,897 persons at enrollment, 66.3% were IgG-positive, and estimated annual FOI was 5.95%. Of 1,269 enrollees participating in all 4 serosurveys, 438 were IgG-negative at enrollment. The annualized FOI ranged from 10% to 20% (during the 2016 outbreak). Overall, we observed high FOI for dengue. These results could support decision-making about control and preventive measures for dengue.

Dengue fever is a mosquitoborne disease caused by 4 related but antigenically distinct dengue viruses (DENVs), serotypes 1–4. Annually, ≈50–100 million cases of dengue are reported worldwide, with 20,000 deaths (*1*). *Aedes* mosquitoes and dengue cases were documented in Africa as early as 1823, and cases have since been reported in 34 countries in Africa (*2*). In Burkina Faso, since the first outbreak in 1925, there have been multiple others (*2*), including in 2013, 2016, and 2017 (*3**–**5*). The 2016 outbreak included 1,061 dengue rapid-diagnostic test (RDT) positive cases and 15 deaths in the capital, Ouagadougou, with a reported case-fatality rate (CFR) of 1.2% (*4*,*6*). The 2017 outbreak included 5,773 RDT-positive cases and 18 deaths throughout the country, for a CFR of 0.2% (*5*). These repeated outbreaks suggest a considerable dengue burden in the country. 

Despite this burden, data on dengue seroprevalence and force of infection (FOI), the rate at which initial or heterotypic infections are acquired, are scarce in Burkina Faso and Africa (*7*). In terms of seroprevalence in Burkina Faso, 1 study found the dengue IgG seroprevalence among 683 pregnant women and blood donors to be 26.3% in rural and 36.5% in urban settings in 2003–2004 (*8*). To define DENV transmission in Burkina Faso, we conducted 4 serologic surveys in the same study participants in Ouagadougou during 2015–2017. The study targeted 3 objectives. First, we measured seroprevalence of DENV by IgG positivity at enrollment, serosurvey 1 (S1). Second, we estimated age-specific annual FOI, measured by seroconversion in the repeated follow-up surveys (S2–S4). Last, because a dengue outbreak occurred in 2016, between the third and fourth serosurveys, we identified and compared demographic and clinical characteristics associated with DENV seroconversion in the outbreak and nonoutbreak periods. 

## Methods

### Study Area and Population

We selected the study area based on data, including seroprevalence and modeling results, available in the literature and existing research infrastructure (*9**–**11*). Ouagadougou is the capital of Burkina Faso in West Africa; most of the population resides in urban settings (*12*,*13*). The rainy season is May–October. The serosurveys were conducted in a defined catchment population of 100,000 residents. The resident population in Ouagadougou is stable, with an annual rate of migration of only 4.1% and >80% of residents owning their homes (*14*). 

### Study Design

We conducted 4 serosurveys, each ≈6 months apart. The age-stratified sample of ≈3,000 residents 1–55 years of age, 80% <35 years of age, reflected the age distribution of the general population of Ouagadougou (*9*). In 6 preselected sectors, we randomly selected households on the basis of existing census data; all eligible household members were offered enrollment. To reach the needed sample size, if members of the initially invited household declined, we invited a neighboring household to enroll. We conducted a short interview and collected blood samples (*9*). Test results were shared with the participants and we followed the same procedures with the same participants for the 3 subsequent serosurveys. 

### Laboratory Testing Algorithm 

We tested samples using a Panbio Dengue IgG Indirect ELISA test (Abbott Diagnostics, https://www.abbott.com), as described elsewhere (*9*). Following the manufacturer’s guidelines, we set the IgG threshold for positivity at an index value of 1.1, to detect levels resulting from past or recent infections of any serotype. An index value of 0.9–1.1 was classified equivocal (requiring repeated testing), and <0.9 was considered negative. We considered seroconversion of dengue IgG between the pretransmission and posttransmission surveys to result from dengue infection. 

### Statistical Analysis 

### Characteristics by Dengue IgG Status at Enrollment

We present a descriptive summary by dengue IgG status at enrollment (seropositive vs. seronegative). We used χ^2^ or Fisher exact tests to make categorical pair-wise comparisons across dengue status. Continuous variables were compared using Student *t*-test or analysis of variance. 

#### FOI Calculations

DENV infection can occur with any of the 4 serotypes and, assuming lifelong acquired homotypic immunity, we estimated the FOI based on IgG seropositivity status (*15*,*16*). However, because IgG ELISA tests cannot distinguish among the 4 serotypes, infection in this analysis refers to seroconversion to any DENV serotype (*15*). We used binomial regression with a complementary log-log link function (*17**–**20*). In part A of the FOI analysis, in which data from the enrollment serosurvey were analyzed, we estimated the average FOI over each participant’s lifetime using age as the time at risk. In part B of the FOI analysis, using data from the subset of participants who contributed to all 4 serosurveys, we estimated the FOI between consecutive surveys. We considered participants who were initially seronegative to be at risk for seroconversion and used the interval between consecutive surveys as the time at risk. We provide details of FOI calculations in the Appendix. 

#### Seroconversion Rate Ratios

For the between-survey analyses, we estimated seroconversion rate ratios (RRs) using binomial regression models with the log time of the actual duration of each participant’s interval (i.e., time between consecutive surveys for that person) for potential risk factors, including age, sex, neighborhood, level of education, occupation, any known previous dengue infection, yellow fever (YF) vaccination history, and any self-reported signs and symptoms during the particular interval. As a sensitivity analysis, seroconversion RRs were estimated for consecutive paired results, irrespective of results from other surveys. For example, if a participant was IgG-negative at S2, then IgG-positive at S3, we considered this seroconversion between S2 and S3, even if the person had been IgG-positive at S1. 

S1–S2 covered the nonoutbreak rainy season in 2015, S2–S3 covered the nonoutbreak nonrainy season in 2016, and S3–S4 covered the 2016 outbreak. To assess how demographic and clinical characteristics are associated with DENV seroconversion and the difference in patterns in the outbreak (S3–S4) compared with those in nonoutbreak periods (S1–S2 and S2–S3), we compiled a descriptive summary of demographic and clinical characteristics for participants at risk (IgG-negative) at each serosurvey, broken down between participants who had or had not seroconverted by the subsequent serosurvey. All analyses were performed using SAS version 9.4 (https://www.sas.com). 

### Ethics Considerations

The study protocol received ethics approvals from the institutional review boards of the International Vaccine Institute, the London School of Hygiene and Tropical Medicine, the National Ethical Committee for Health Research of Burkina Faso, and the Ethics Committee of the Centre Hospitalier de l'Université de Montréal at University of Montreal. We obtained written consent forms from each participant >18 years old. For participants 8–17 years old, we obtained an assent form from the participant and an informed consent from >1 parent or legal guardian. For participants ≤7 years old, we obtained an informed consent from >1 parent or legal guardian. 

## Results

**Figure 1:**
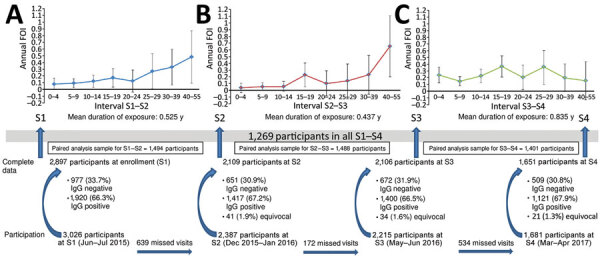
Flowchart of participant enrollment and graphs of annual force of infection rates during a rainy season (A), a nonrainy season (B), and the 2016 dengue outbreak (C) in study of force of infection for dengue virus, Ouagadougou, Burkina Faso, June–July 2015. Labels along x-axes (e.g., 0–4) indicate age ranges in years. Error bars indicate 95% CIs. To be considered complete, records had to contain lab results and basic demographic information. Seroconversion rates in the paired analyses were based solely on results from one survey to the next (e.g., S2–S3). Dengue serostatus in previous or subsequent surveys was not considered. S, serosurvey.

We obtained complete demographic (age and neighborhood) and laboratory data for 2,897 of 3,026 participants (Figure 1). At enrollment (S1), in June 2015, 1,920 (66.3%) of 2,897 participants were IgG positive. At S2, in December 2015, 1,417 (67.2%) of 2,109 participants were IgG positive. At S3, in May 2016, 1,400 (66.5%) of 2,106 participants were IgG positive. At S4, in March 2017, 1,121 (67.9%) of 1,651 participants were IgG positive. 

### Characteristics by Dengue IgG Status at Enrollment

**Figure 2:**
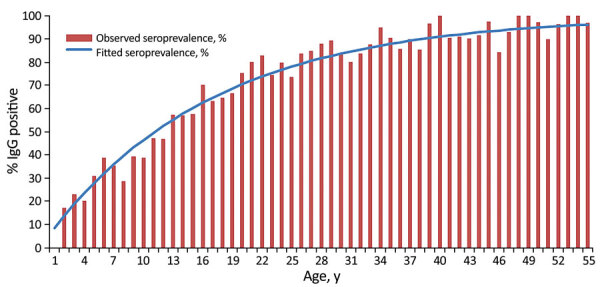
Seroprevalence, measured by IgG ELISA, of dengue IgG by age at enrollment and fitted prevalence using the FOI per year in study of force of infection for dengue virus, Ouagadougou, Burkina Faso, June–July 2015. Graph shows observed seroprevalence at enrollment among all 2,897 participants and fitted seroprevalence using FOI. In the FOI analysis part A, the FOI per year was 0.0595 (95% CI 0.0566–0.0624), estimated by binomial regression, with the assumption of constant risk across ages and calendar time prior to the enrollment serosurvey, and a complementary log-log link, with log(midpoint of age) as an offset. The intercept is interpreted as the logarithm of the FOI. FOI, force of infection.

Based on data from the 2,897 participants with complete information, 82% of 1–4-year-olds and 65% of 5–9-year-olds were IgG negative and therefore at risk of infection at the start of the study (Table 1). IgG positivity increased with age, so that by age 26, IgG positivity reached 80% (Figure 2). The binomial regression based on IgG positivity by age at enrollment, assuming the FOI was constant over ages and calendar time (part A), resulted in an annual FOI of 5.95% (95% CI 5.66–6.24) (Figure 2). 

**Table 1:** Demographic and clinical characteristics of participants in study of force of infection for dengue virus, by dengue IgG status at enrollment, Ouagadougou, Burkina Faso, June–July 2015*

Characteristics	All participants,† N = 2,897	Seropositive,‡ n = 1,920	Seronegative,‡ n = 977	p value§
Mean age, SD	22.32 (13.92)	27.02 (13.36)	13.08 (9.74)	<0.001
Age group, y				<0.001
1–4	208 (7.2)	37 (1.9)	171 (17.5)	
5–9	410 (14.2)	142 (7.4)	268 (27.4)	
10–14	384 (13.3)	189 (9.8)	195 (20.0)	
15–19	379 (13.1)	243 (12.7)	136 (13.9)	
20–29	694 (24.0)	560 (29.2)	134 (13.7)	
30–39	410 (14.2)	357 (18.6)	53 (5.4)	
40–49	264 (9.1)	249 (13.0)	15 (1.5)	
50–55	148 (5.1)	143 (7.5)	5 (0.5)	
Sex¶				
M	1,154 (39.9)	700 (36.5)	454 (46.5)	<0.001
F	1,741 (60.1)	1,218 (63.5)	523 (53.5)	
Ethnicity				0.712
Burkinabé	2,871 (99.6)	1,906 (99.6)	965 (99.5)	
Others	13 (0.5)	8 (0.4)	5 (0.5)	
Neighborhood/sector				<0.001
Sector 22	447 (18.2)	342 (20.5)	105 (13.3)	
Sector 25	510 (20.7)	395 (23.6)	115 (14.6)	
Juvenat fille	517 (21.0)	353 (21.1)	164 (20.8)	
Pazani	433 (17.6)	281 (16.8)	152 (19.2)	
Zongo	547 (22.2)	297 (17.8)	250 (31.7)	
Nioko	8 (0.3)	4 (0.2)	4 (0.5)	
Occupation				<0.001
Student	1,033 (35.8)	586 (30.6)	447 (46.0)	
Housewife	885 (30.7)	666 (34.8)	219 (22.5)	
Small business owner	163 (5.7)	130 (6.8)	33 (3.4)	
Unskilled worker	153 (5.3)	124 (6.5)	29 (3.0)	
Government official	92 (3.2)	77 (4.0)	15 (1.5)	
Private sector employee	82 (2.8)	66 (3.5)	16 (1.7)	
Merchant	55 (1.9)	46 (2.4)	9 (0.9)	
Retired	53 (1.8)	26 (1.4)	27 (2.8)	
Farmer	49 (1.7)	35 (1.8)	14 (1.4)	
Skilled worker	43 (1.5)	32 (1.7)	11 (1.1)	
Service sector worker	43 (1.5)	34 (1.8)	9 (0.9)	
Education level				<0.001
Illiterate	887 (30.7)	596 (31.1)	291 (29.9)	
Literate, no education	72 (2.5)	58 (3.0)	14 (1.4)	
1–6 y of school	803 (27,8)	455 (23.8)	348 (35.8)	
7–10 y of school	551 (19.1)	388 (20.3)	163 (16.8)	
11–13 y of school	274 (9.5)	200 (10.4)	74 (7.6)	
>University	210 (7.5)	171 (8.9)	39 (3.0)	
Others#	57 (2.0)	39 (2.0)	18 (1.9)	
Self-reported preexisting conditions				
Cardiovascular	113 (4.0)	100 (5.3)	13 (1.4)	<0.001
Diabetes	10 (0.4)	7 (0.4)	3 (0.3)	0.817
Lung disease	19 (0.7)	17 (0.9)	2 (0.2)	0.034
Cerebrovascular	27 (0.9)	18 (0.9)	9 (0.9)	0.990
Musculoskeletal	101 (3.5)	82 (4.3)	19 (2.0)	0.002
Gastrointestinal	193 (6.7)	148 (7.8)	45 (4.7)	0.002
Anemia	10 (0.4)	2 (0.1)	8 (0.80)	0.002
Others	108 (3.8)	79 (4.1)	29 (3.0)	0.139
Self-reported previous dengue				<0.001
Yes	13 (0.5)	13 (0.7)	0	
No	2,421 (84.7)	1,635 (86.0)	786 (82.1)	
Unknown	426 (14.9)	254 (13.4)	172 (18.0)	

*Values are no. (%) except as indicated.  †Total participants enrolled at serosurvey 1. ‡Results of IgG indirect ELISA among participants at serosurvey 1. §p values based on χ^2^ test.  ¶2 individuals with missing information on sex. #Religious and other informal education.

### Annual FOI in IgG-Negative Participants Who Contributed to All 4 Serosurveys

In part B, we calculated annualized FOI among participants who had been IgG negative at the preceding serosurvey using binomial regression with the intersurvey interval, calculated in years, as the time at risk. For the interval S1–S2, FOI per year was 14.0% (95% CI 9.5%–18.4%); for the interval S2–S3, 9.6% (95% CI 5.4%–13.8%); and for the interval S3–S4, 20.3% (95% CI 16.1%–24.5%) (Figure 1). The mean duration was 0.53 years (≈6 months) for interval S1–S2; 0.44 years for S2–S3; and 0.84 years for S3–S4. Age-specific annual FOIs were calculated and older age groups with <5 seronegative participants were merged. For intervals S1–S2 and S2–S3, FOI was higher for older participants (Figure 1). For interval S3–S4, high FOIs of 30% per year were found in participants 15–19 and 25–29 years of age. 

### Seroconversion RRs from Outbreak Versus Nonoutbreak Intervals

We analyzed data from each pair of surveys, not restricted to data from participants in all 4 surveys, to examine differences in dengue seroconversion during outbreak versus nonoutbreak intervals. During nonoutbreak interval S1–S2 (analysis sample = 1,494), 33 (7.3%) of 455 participants at risk (i.e., IgG negative at S1), showed seroconversion. During nonoutbreak interval S2–S3 (analysis sample = 1,488), 23 (5.2%) of 443 participants at risk (i.e., IgG negative at S2), showed seroconversion. During outbreak interval S3–S4 (analysis sample = 1,401) 78 (17.1%) of 455 participants at risk (i.e., IgG negative at S3), showed seroconversion. We compiled demographic and clinical characteristics of participants with IgG seroconversion compared with participants who remained IgG negative during each interval (Table 2). 

**Table 2:** Demographic and clinical characteristics of participants in study of force of infection for dengue virus, by status of IgG seroconversion in paired surveys, Ouagadougou, Burkina Faso, June–July 2015*

Characteristics	S1–S2,† N = 455					S2–S3,† N = 443					S3–S4,† N = 455			
	All	IgG-S,‡ n = 33	IgG-N,‡ n = 422	p value§		All	IgG-S, ‡ n = 23	IgG-N,‡ n = 420	p value§		All	IgG-S, ‡ n = 78	IgG-N,‡ n = 377	p value§
Age range, y				**0.003**					**0.002**					**0.037**
1–4	77	4 (5.2)	73 (94.8)			74	1 (1.4)	73 (98.7)			78	13 (16.7)	65 (83.3)	
5–9	138	5 (3.6)	133 (96.4)			138	3 (2.2)	135 (97.8)			146	16 (11.0)	130 (89.0)	
10–14	97	5 (5.2)	92 (94.9)			95	4 (4.2)	91 (95.8)			97	16 (16.5)	81 (83.5)	
15–24	91	9 (9.9)	82 (90.1)			85	7 (8.2)	78 (91.8)			85	23 (27.1)	62 (72.9)	
25–55	52	10 (19.2)	42 (80.8)			51	8 (15.7)	43 (84.3)			49	10 (20.4)	39 (79.6)	
Sex				0.160					0.169					0.733
M	219	12 (5.5)	207 (94.5)			216	8 (3.7)	208 (96.3)			218	36 (16.5)	182 (83.5)	
F	236	21 (8.9)	215 (91.1)			227	15 (6.6)	212 (93.4)			237	42 (17.7)	195 (82.3)	
Neighborhood				0.097					0.118					0.072
Juvenat fille	103	5 (4.9)	98 (95.2)			100	3 (3.0)	97 (97.0)			94	24 (25.5)	70 (74.5)	
Nioko	4	0	4 (100.0)			4	0	4 (100.0)			2	0	2 (100.0)	
Pazani	73	11 (15.1)	62 (84.9)			63	0	63 (100.0)			68	12 (17.7)	56 (82.4)	
Zongo	118	6 (5.1)	112 (94.9)			121	10 (8.3)	111 (91.7)			157	17 (10.8)	140 (89.2)	
Sector 22	61	3 (4.9)	58 (95.1)			62	3 (4.8)	59 (95.2)			57	12 (21.1)	45 (79.0)	
Sector 25	96	8 (8.3)	88 (91.7)			93	7 (7.5)	86 (92.5)			77	13 (16.9)	64 (83.1)	
Preexisting conditions¶				**0.014**					**0.013**					0.458
No/unknown	404	25 (6.2)	379 (93.8)			396	17 (4.3)	379 (95.7)			402	67 (16.7)	335 (83.3)	
Yes	51	8 (15.7)	43 (84.3)			47	6 (12.8)	41 (87.2)			53	11 (20.8)	42 (79.3)	
Occupation				0.930					**0.016**					0.180
Student	221	15 (6.8)	206 (93.2)			212	6 (2.8)	206 (97.2)			232	34 (14.7)	198 (85.3)	
At home#	106	8 (7.6)	98 (92.5)			106	11 (10.4)	95 (89.6)			105	24 (22.9)	81 (77.1)	
Others**	128	10 (7.8)	118 (92.2)			125	6 (4.8)	119 (95.2)			118	20 (17.0)	98 (83.1)	
Level of education				0.921					0.170					0.159
Illiterate/no schooling	138	9 (6.5)	129 (93.5)			139	11 (7.9)	128 (92.1)			136	23 (16.9)	113 (83.1)	
Elementary	169	13 (7.7)	156 (92.3)			161	5 (3.1)	156 (96.9)			182	25 (13.7)	157 (86.3)	
>Secondary	148	11 (7.4)	137 (92.6)			143	7 (4.9)	136 (95.1)			137	30 (21.9)	107 (78.1)	
YF vaccination¶				0.371					0.469					0.535
No/unknown	445	33 (7.4)	412 (92.6)			429	23 (5.4)	406 (94.6)			427	72 (16.9)	355 (83.1)	
Yes	10	0	10 (100.0)			14	0	14 (100.0)			28	6 (21.4)	22 (78.6)	
Previous dengue¶				NA					0.052					0.865
No/unknown	455	33 (7.3)	422 (92.8)			442	22 (5.0)	420 (95.0)			450	77 (17.1)	373 (82.9)	
Yes	0	0	0			1	1 (100.0)	0			5	1 (20.0)	4 (80.0)	
Signs and symptoms¶														
Fever	192	15 (7.8)	177 (92.2)	0.694		111	5 (4.5)	106 (95.5)	0.706		218	41 (18.8)	177 (81.2)	0.366
Fatigue, weakness	126	9 (7.1)	117 (92.9)	0.955		108	6 (5.6)	102 (94.4)	0.845		158	35 (22.2)	123 (77.9)	0.039
Retro-orbital pain	34	4 (11.8)	30 (88.2)	0.295		26	2 (7.7)	24 (92.3)	0.637		16	1 (6.3)	15 (93.8)	0.239
Headache	248	22 (8.9)	226 (91.1)	0.145		230	9 (3.9)	221 (96.1)	0.207		292	51 (17.5)	241 (82.5)	0.807
Rash	23	0	23 (100.0)	0.397		29	2 (6.9)	27 (93.1)	0.656		18	4 (22.2)	14 (77.8)	0.560
Eye pain	39	5 (12.8)	34 (87.2)	0.161		20	3 (15.0)	17 (85.0)	0.078		28	5 (17.9)	23 (82.1)	0.918
Arthralgia	81	8 (9.9)	73 (90.1)	0.315		79	8 (10.1)	71 (89.9)	0.029‡		81	14 (17.3)	67 (82.7)	0.970
Myalgia	57	8 (14.0)	49 (86.0)	0.035		60	3 (5.0)	57 (95.0)	0.943		65	15 (23.1)	50 (76.9)	0.170
Diarrhea	52	6 (11.5)	46 (88.5)	0.206		57	1 (1.8)	56 (98.3)	0.338		40	3 (7.5)	37 (92.5)	0.090
Nausea, vomiting	111	11 (9.9)	100 (90.1)	0.215		117	4 (3.4)	113 (96.6)	0.466		102	11 (10.8)	91 (89.2)	0.053
Abdominal pain	123	10 (8.1)	113 (91.9)	0.661		155	7 (4.5)	148 (95.5)	0.638		180	25 (13.9)	155 (86.1)	0.136
Appetite loss	128	13 (10.2)	115 (89.8)	0.135		179	5 (2.8)	174 (97.2)	0.061		114	23 (20.2)	91 (79.8)	0.321
Neck pain	18	2 (11.1)	16 (88.9)	0.381		17	3 (17.7)	14 (82.4)	0.052		14	1 (7.1)	13 (92.9)	0.481
Sore throat	30	3 (10.0)	27 (90.0)	0.470		28	2 (7.1)	26 (92.9)	0.649		21	3 (14.3)	18 (85.7)	0.722
Nasal congestion	73	4 (5.5)	69 (94.5)	0.630		58	1 (1.7)	57 (98.3)	0.339		74	9 (12.2)	65 (87.8)	0.214
Cough	72	6 (8.3)	66 (91.7)	0.700		71	1 (1.4)	70 (98.6)	0.149		115	19 (16.5)	96 (83.5)	0.838

*Values are no. or no. (%) except as indicated. Bold indicates statistical significance. IgG-S, IgG seroconverted; IgG-N, IgG negative; NA, not available; S, serosurvey; YF, yellow fever.  †Paired survey intervals. S1–S2: July–December 2015; S2–S3: January 2016–May 2016; S3–S4: June 2016–March 2017. ‡Results of IgG indirect ELISA. §p values based on χ^2^ test. ¶Based on self-report by participants. #Housewife, retired, unemployed. **Business owners, employees, workers, etc.

To assess how these variables might be associated with changes in rates of seroconversion, we estimated RRs of seroconversion during the intervals. We found that older age was positively associated with an increased rate of seroconversion. Compared with those 1–4 years of age, participants 25–55 years of age had higher seroconversion over S1–S2 [RR 4.1 (95% CI 1.4–15.0)]; both 15–24-year-old participants [RR 4.6 (95% CI 1.4–17.4)] and 25–55-year-old participants [RR 9.1 (95% CI 12.9–34.2)] had higher seroconversion over S2–S3 (Table 3). 

**Table 3:** Univariable binomial regression showing ratio of rates of seroconversion in study of force of infection for dengue virus, based on results of IgG indirect ELISA assays, Ouagadougou, Burkina Faso, June–July 2015*

Characteristics	S1–S2,† N = 455, IgG-S (n = 33) vs. IgG-N (n = 422)			S2–S3,† N = 443, IgG-S (n = 23) vs. IgG-N (n = 420)			S3–S4,† N = 455, IgG-S (n = 78) vs. IgG-N (n = 377)	
	IgG-S rate (95% CI)	p value‡		IgG-S rate (95% CI)	p value‡		IgG-S rate (95% CI)	p value‡
Age range, y		**0.006**			**0.002**			0.038
1–4	Referent			Referent§			Referent	
5–9	0.70 (0.19–2.84)						0.65 (0.31–1.37)	
10–14	1.00 (0.27–4.05)			2.25 (0.53–9.52)			1.00 (0.48–2.12)	
15–24	1.99 (0.65–7.35)			**4.55 (1.37–17.38)**			1.77 (0.91–3.60)	
25–55	**4.11 (1.37–14.99)**			**9.13 (2.87–34.20)**			1.29 (0.55–2.94)	
Sex		0.155			0.166			0.686
M	Referent			Referent			Referent	
F	1.67 (0.84–3.51)			1.83 (0.80–4.55)			1.10 (0.70–1.72)	
Preexisting conditions¶		**0.016**			**0.017**			0.433
None/unknown	Referent			Referent			Referent	
Yes	**2.67 (1.13–5.67)**			**3.11 (1.12–7.48)**			1.29 (0.64–2.34)	
Occupation		0.935			**0.025**			0.177
Student	Referent			Referent			Referent	
At home#	1.11 (0.45–2.56)			**3.81 (1.45–11.05)**			1.64 (0.96–2.75)	
Others**	1.15 (0.50–2.54)			1.70 (0.53–5.45)			1.16 (0.66–2.01)	
Level of education		0.917			0.191			0.164
Illiterate/no schooling	Referent			Referent			Referent	
Elementary	1.19 (0.52–2.89)			0.39 (0.12–1.06)			0.80 (0.45–1.42)	
>Secondary	1.15 (0.48–2.85)			0.61 (0.23–1.55)			1.33 (0.78–2.32)	
*Bold indicates statistical significance. IgG-S, IgG seroconverted; IgG-N, IgG negative; S, serosurvey. †Paired survey intervals. S1–S2: July–December 2015; S2–S3: January 2016–May 2016; S3–S4: June 2016–March 2017. ‡p values based on χ^2^ test. §Age groups 1–4 and 5–9 merged due to data scarcity in seroconverted participants. ¶Based on self-report by participants. #Housewife, retired, unemployed. **Business owners, employees, workers, etc.								

## Discussion

Our study provides data on population-based seroprevalence and rates of DENV seroconversion that may help with assessing the largely undocumented burden of dengue fever in Africa. The dengue burden in Africa has been conjectured to be similar to that in the Americas but has been largely unrecognized and masked by other illnesses with similar symptoms (*21*,*22*). In particular, the major strength of this study was that we longitudinally followed the same participants using 4 repeated serosurveys to measure the FOI of dengue in Burkina Faso. 

Although IgG ELISA test results might be influenced by cross-reactivity with different flaviviruses, our estimate of seroprevalence was comparable to prevalence estimates from other studies, all tested using IgG ELISA: 61% of participants 1–65 years of age in Colombia (*25*); 83.1% in participants 15–19 years of age in Tahiti (*26*); 74% in participants in a low socioeconomic area in Recife, Brazil (*27*); and 68.7% in participants in Salvador, Brazil (*28*). The overall proportion of IgG-positive participants remained similar across the surveys, although it was highest at S4 (68%). The small increase in seropositivity despite an outbreak between S3 and S4 may have resulted from different participants being lost to follow-up at each consecutive survey. Such losses were substantial during the S1–S2 and S3–S4 intervals. The mean age of participants lost during S1–S2 was 20.7 years (95% CI 19.3–22.0) and was 23.7 (95% CI: 22.5–24.9) during S3–S4. The mean age of the participants was 22.2 years (95% CI 21.6–22.9) during S1–S2 and 21.6 (95% CI 21.0–22.2) during S3–S4. For S1–S2, the mean age was not significantly different between nonparticipants and remaining participants, but lost participants were significantly older for S3–S4; participants lost at follow-up might have tended to be IgG positive, plausibly resulting in underestimating IgG positivity at the follow-up survey. While decreasing representativeness is a limitation, we based FOI calculations on seroconversion meaning they likely would not have been substantially affected. We found apparent seroreversion from seropositive to seronegative in <3% of the paired samples: 29 during S1–S2, 14 during S2–S3; and 38 during S3–S4. However, we could not distinguish whether such cases were due to test errors or waning immunity. 

Data from the baseline serosurvey in this study (part A) resulted in an annual FOI of 6.0%, although this assumes that it was constant over age and calendar time. This finding is comparable with estimates from other regions, such as Sri Lanka at 14% per year (*15*); Colombia at 8.7% (*22*); and a low socioeconomic area in Recife, Brazil, at 5.3% (*25*). Our model seems to capture the increase in baseline seroprevalence with age (Figure 1). However, any given age profile in a cross-sectional study can result from different combinations of incidence varying over age or over calendar time (*27*). Therefore, the estimated FOI from the baseline survey is subject to more limitations than FOIs estimated from the paired surveys. Nonetheless, our data support that dengue may be a bigger public health problem in Africa than is currently recognized. 

From the repeat surveys (part B) for which we knew the exact duration of the intervals, we could measure the FOI more directly without assuming a constant value over age, providing a more accurate estimate of the magnitude of transmission. These estimates were higher than the 6% annual FOI based on the enrollment survey. Among 1,269 participants with IgG results from all 4 serosurveys, annual FOIs were 14% for S1–S2, 10% for S2–S3, and 20% for S3–S4. Testing of paired samples showed that 7.3% (33/455) seroconverted during S1–S2, 5.2% (23/443) during S2–S3, and 17.1% (78/455) during S3–S4. As we expected, the annualized estimates of rates were ≈2 times the simple proportions for the first 2 intervals. For the third interval (S3–S4), the annualized FOI was ≈1.5 times (12/8 months) the proportion seroconverting, because the conversion between proportions and rates is less linear for higher values. 

The paired survey analyses did not require an assumption of constant FOI before the study and showed that FOI varied markedly across time within the study. In particular, a high annualized FOI (20%) was observed between S3–S4, coinciding with a major outbreak. However, even during nonoutbreak intervals, we found considerable dengue transmission; annualized FOIs ranged from 10% (in a nonrainy season, such as S2–S3) to 14% (in a rainy season, S1–S2) (*28*). Little comparable information from the region is available, but these rates are comparable to the 8.7% per year reported from Colombia (*23*). 

These annual rates of infection, measured by seroconversion, may include both initial and subsequent heterotypic infection, because these are indistinguishable by IgG ELISA. However, in our analysis, the influence of past infection is minimal, because we focused on participants with IgG-negative status at enrollment for calculations of FOIs between surveys. This study was unable to distinguish participants with single or multiple infections during the study period. Furthermore, among those with multiple infections, cyclical dominance among DENV serotypes is observed in regions with better-documented dengue endemicity (*29*,*30*). However, cocirculation of multiple DENV serotypes in Burkina Faso has yet to be fully demonstrated. Thus, in our study, we calculated an overall FOI, for the totality of serotypes (*20*,*31*). Still, we recognize the need for more in-depth analyses using neutralization assays to understand serotype-specific transmission patterns in Africa. 

In part B of the study, without having to assume that risk is constant over age, we found that FOI was higher in older than in younger participants, although these findings are based on small numbers, given that most people had already seroconverted at younger ages. FOI increased with age in the intervals of the nonoutbreak times (S1–S2 and S2–S3), which has also been found in Colombia (*23*). Our data are from IgG seroconversion rather than clinical dengue, but incidence of dengue fever in the 2016 outbreak in Burkina Faso was also higher among teenagers and young adults (*4*,*6*,*32*). In contrast, in the outbreak interval S3–S4, the FOI was similar across ages (13%–34%), possibly suggesting the emergence of a different serotype with little preexisting population immunity (*20*). Without much data on serotype-specific DENV incidence in Burkina Faso, previous studies and reports of ministry of health and World Health Organization investigations suggest that the 2016 outbreak was mainly caused by DENV-2 (*4*,*6*,*32*).

Myalgia during S1–S2, arthralgia during S2–S3, and fatigue during S3–S4 were positively associated with seroconversion with statistical significance. Whereas cases of seroconversion may have been associated with either mild or subclinical illnesses, participants were asked whether they had experienced these symptoms in the interval since the previous survey. These symptoms are common in dengue illness; myalgia and arthralgia were listed in the 1997 World Health Organization dengue case classifications, and fatigue or lethargy in 2009 classifications (*33**–**35*). However, because these self-reported symptoms were recorded at intervals of 6–8 months, without prompt investigation to identify a cause, we cannot conclude that any associations with seroconversion are causal. 

This study is subject to several limitations. First, the generalizability of this study is limited by participants all being recruited from the urban population in the capital city. The magnitude and patterns of DENV transmission may differ in other regions of Burkina Faso, including rural areas, with different age profiles, ecologic settings, or socioeconomic conditions. Also, although a large number of participants (1,269) participated in all 4 serosurveys, a substantial number of original participants missed surveys. Those participants who missed >1 surveys were more likely to be older than those who participated in all surveys. This shortfall could have led us to underestimate prevalence. 

Second, our results were based on serologic testing using IgG ELISA. Further analyses using neutralization assays are planned, but no confirmatory testing was applied to verify the IgG results. For estimating dengue FOI, serologic cross-reaction with other flaviviruses affecting the observed dengue IgG rates is a commonly reported limitation (*36*,*37*). Ultimately, this limitation could result in inaccurate seroprevalence and FOI estimates because antibodies against nondengue flaviviruses could have been detected (*15*,*38*). In particular, Zika virus has been reported in Burkina Faso (*39*,*40*), as have outbreaks of YF in 1998, 2003, and 2004 (*41**–**43*). Vaccine-induced YF antibodies could also result in interference with the specificity of IgG ELISA (*38*). In our data, at each of the serosurveys, <5% of participants answered that they had received YF vaccination; although subject to recall bias, this rate is much lower than the coverage rate of 85% reported by the Expanded Program on Immunization in 2007 (*42*). Using self-reported YF vaccination history as a proxy for YF virus seropositivity, we found no difference in IgG positivity between participants reporting and not reporting vaccination, a finding supported by others (*38*,*44*). 

Despite the possible effect of cross-reactivity on dengue ELISAs, 2 studies reported a concordance level of 99% between the plaque reduction neutralization test (PRNT) and dengue IgG results (*37*,*45*). Also, when IgG ELISA results were verified by using serologic data among 13,661 participants from 13 countries to estimate dengue FOI, IgG ELISA results were confirmed by 50% PRNT in >97% of the IgG-positive samples (*20*). Samples from 277 healthy adults in a rural district in Malaysia were tested with the same ELISA test used in our study, and PRNT was performed on a subset of IgG-positive samples (*46*). Evidence of past infection was found in 75.5% (209/277) of participants and, of 96 samples randomly selected to undergo PRNT testing, all 96 were dengue-confirmed with >50% plaque reduction for DENV and showed that the detected antibodies were specific to dengue virus (*46*). 

The sensitivity of this commercial DENV IgG indirect ELISA was reported as 99.2% and the specificity as 96.2% when compared with the hemagglutination inhibition (HI) method (*47*). A study compared performance of IgG ELISA to that of HI testing in a serosurvey of 327 children in Tahiti and reported sensitivity and specificity, as well as agreement between the 2 tests, to be >83% for all DENV serotypes (*24*). Another study reported 90.9% sensitivity and 92.9% specificity of the IgG indirect ELISA when compared with HI, also with a high correlation between the tests (*48*). Whereas those data are from outside of Africa, where a different and unknown composition of flaviviruses may be circulating, the results indicate a high degree of agreement between IgG ELISA and more confirmatory and better validated tests, such as HI and PRNT. 

If cross-reactivity across other flaviviruses were to result in misclassifications, leading to a high rate of false positives, our findings would be overestimates of the true disease prevalence and FOI. However, given the high concordance between IgG and PRNT or HI assay results based on available data and because we analyzed data from paired serosurveys of participants who were IgG negative at enrollment, we believe that our results were minimally affected by this issue. 

In conclusion, our estimates of both seroprevalence and FOI were comparable with those from dengue-endemic countries in the Americas. Repeated outbreaks indicate a considerable level of DENV transmission in Ouagadougou, but the extent of transmission and hyperendemicity needs to be further verified. Specifically, additional longitudinal evaluation with confirmatory tests and linked clinical evaluation of dengue fever in the general population in the region would be necessary to further validate our findings. Seroprevalence and FOI are important factors to be considered when making evidence-based decisions to implement interventions for prevention and control, including vaccine introduction. In the absence of other reliable data, our findings on dengue seroprevalence and FOI based on consecutive serosurveys provide practical evidence that could be used to support policy decisions. 

Appendix Additional details of methodology used in study of force of infection for dengue virus, Ouagadougou, Burkina Faso, June–July 2015.
