# Supplementary material for: Estimating the Force of Infection for Dengue Virus Using Repeated Serosurveys, Ouagadougou, Burkina Faso
Source: Emerg Infect Dis. 2021 Jan;27(1):130–9. doi: 10.3201/eid2701.191650 (PMC7774580; doi:10.3201/eid2701.191650)
Supplement: Appendix — Additional details of methodology used in study of force of infection for dengue virus, Ouagadougou, Burkina Faso, June–July 2015. [file 19-1650-Techapp-s1.pdf]

Article DOI: https://doi.org/10.3201/eid2701.191650

Estimating the Force of Infection for Dengue Virus Using Repeated Serosurveys, Ouagadougou, Burkina Faso

Appendix

Force of Infection Calculation in IgG-Negative Subjects Who Contributed to All 4 Serosurveys

Assuming a constant rate of exposure (μ) to the totality of serotypes, with μ being the force of infection (FOI), the probability of a participant seroconverting within the time, t, to the subsequent serosurvey is $1\text{-}e^{-\mu t}$ (*1*). If μ is assumed constant across ages and calendar time before the enrollment serosurvey, then if participant i has age A_i at that serosurvey, t equals A_i. So, if this participant's probability of being seropositive is denoted p_i then $\log[-\log(1-p_i)] = \log(\mu) + \log(A_i)$ (*1*).

Using data from the subset of subjects who contributed to all 4 serosurveys, we used binomial regression with a complementary log-log link function, as reflected in the left-hand side of the equation above (*1*–*6*). First, from the baseline seroprevalence survey (FOI analysis part A), we estimated the average FOI over each participant's lifetime by taking their age as the time at risk, including the logarithm of age in the model as an offset. Then, in accordance with the above equation, the intercept of this model estimates the logarithm of the FOI (*1*). In this analysis, the baseline seroprevalence survey was analyzed alone, subject to the above assumption of constant FOI over age or calendar time, as has been done in comparable studies (*4*,*7*,*8*).

Then, to estimate FOI between consecutive surveys, we considered those participants who were initially seronegative to be at risk of seroconversion, with the interval between surveys used as the time at risk (FOI analysis part B). This between-survey analysis does not require the FOI to be constant over age. Rather, age can be included among other risk factors in the regression, and seroconversion rate ratios obtained. The logarithm of the time between surveys, instead of the logarithm of age, is included as an offset.

References

1. Collett D. Modelling binary data. London: Chapman and Hall; 1991.

2. Bhavsar A, Tam CC, Garg S, Jammy GR, Taurel AF, Chong SN, et al. Estimated dengue force of infection and burden of primary infections among Indian children. BMC Public Health. 2019;19:1116. PubMed https://doi.org/10.1186/s12889-019-7432-7

3. Prayitno A TA, Nealon J, Satari HI, Karyanti MR, Sekartini R, Soedjatmiko S, Gunardi H, Medise BE, Sasmono RT, Simmerman JM, Bouckenooghe A, Hadinegoro SR. Dengue seroprevalence and force of primary infection in a representative population of urban dwelling Indonesian children. PLoS Negl Trop Dis. 2017;11:e0005621. PubMed https://doi.org/10.1371/journal.pntd.0005621. Erratum in: PLoS Negl Trop Dis. 2018;12:e0006467.

4. Nealon J, Bouckenooghe A, Cortes M, Coudeville L, Frago C, Macina D, et al. Dengue endemicity, force of infection, and variation in transmission intensity in 13 endemic countries. J Infect Dis. 2020:jiaa132. PubMed https://doi.org/10.1093/infdis/jiaa132. Erratum in: J Infect Dis. 2020;222:341–2.

5. Arnold D. PROBIT and complementary log-log models. Edmonton, Alberta, Canada: University of Alberta; 2004.[cited 2019 May 1]. http://www.stat.ualberta.ca/~kcarrier/STAT562/daniel.pdf

6. Agresti A. Categorical data analysis. 3rd ed. Toronto: Wiley; 2012.

7. Ferguson N, Donnelly C, Anderson R. Transmission dynamics and epidemiology of dengue: insights from age-stratified sero-prevalence surveys. Philos Trans R Soc Lond B Biol Sci. 1999;354:757–68. PubMed https://doi.org/10.1098/rstb.1999.0428

8. Katzelnick LC, Ben-Shachar R, Mercado JC, Rodriguez-Barraquer I, Elizondo D, Arguello S, et al. Dynamics and determinants of the force of infection of dengue virus from 1994 to 2015 in Managua, Nicaragua. Proc Natl Acad Sci U S A. 2018;115:10762–7. PubMed https://doi.org/10.1073/pnas.1809253115

Appendix Table. STROBE Statement. Force of infection of dengue virus estimated using repeated serosurveys in Ouagadougou, Burkina Faso*†

Content	Item no.	Recommendation
Title and abstract	1	(*a*) Indicate the study's design with a commonly used term in the title or the abstract (title on Page 1)<br>(*b*) Provide in the abstract an informative and balanced summary of what was done and what was found (abstract on Page 3)
Introduction		
Background/ rationale	2	Explain the scientific background and rationale for the investigation being reported (background, para 2-4)
Objectives	3	State specific objectives, including any prespecified hypotheses (background, para 5)
Methods		
Study design	4	Present key elements of study design early in the paper (methods, para 3-5)
Setting	5	Describe the setting, locations, and relevant dates, including periods of recruitment, exposure, follow-up, and data collection (methods para 1-2)
Participants	6	(*a*) *Cohort study*—Give the eligibility criteria, and the sources and methods of selection of participants. Describe methods of follow-up<br>*Case-control study*—Give the eligibility criteria, and the sources and methods of case ascertainment and control selection. Give the rationale for the choice of cases and controls<br>*Cross-sectional study*—Give the eligibility criteria, and the sources and methods of selection of participants (methods, para 4-5)<br>(*b*) *Cohort study*—For matched studies, give matching criteria and number of exposed and unexposed<br>*Case-control study*—For matched studies, give matching criteria and the number of controls per case
Variables	7	Clearly define all outcomes, exposures, predictors, potential confounders, and effect modifiers. Give diagnostic criteria, if applicable (methods, para 6-7, 9-10)
Data sources/ measurement	8	For each variable of interest, give sources of data and details of methods of assessment (measurement). Describe comparability of assessment methods if there is more than one group (methods, para 10-13)
Bias	9	Describe any efforts to address potential sources of bias (methods, para 9, 12)
Study size	10	Explain how the study size was arrived at (Figure 1)
Quantitative variables	11	Explain how quantitative variables were handled in the analyses. If applicable, describe which groupings were chosen and why (specified in the tables)
Statistical methods	12	(*a*) Describe all statistical methods, including those used to control for confounding (methods para 8, 10-13)<br>(*b*) Describe any methods used to examine subgroups and interactions (-)<br>(*c*) Explain how missing data were addressed (results para 1)<br>(*d*) *Cohort study*—If applicable, explain how loss to follow-up was addressed<br>*Case-control study*—If applicable, explain how matching of cases and controls was addressed<br>*Cross-sectional study*—If applicable, describe analytical methods taking account of sampling strategy (Not applicable)<br>(*e*) Describe any sensitivity analyses (methods para 12)
Results		
Participants	13	(a) Report numbers of individuals at each stage of study—e.g., numbers potentially eligible, examined for eligibility, confirmed eligible, included in the study, completing follow-up, and analyzed (figure 1, results para 1)<br>(b) Give reasons for non-participation at each (figure 1, results para 1)<br>(c) Consider use of a flow diagram (figure 1)
Descriptive data	14	(a) Give characteristics of study participants (e.g., demographic, clinical, social) and information on exposures and potential confounders (table 1, results para 2)<br>(b) Indicate number of participants with missing data for each variable of interest (figure 1)<br>(c) *Cohort study*—Summarize follow-up time (e.g., average and total amount) (Not applicable)
Outcome data	15	*Cohort study*—Report numbers of outcome events or summary measures over time<br>*Case-control study*—Report numbers in each exposure category, or summary measures of exposure<br>*Cross-sectional study*—Report numbers of outcome events or summary measures (figure 1, Tables 1, 2, 3, results para 3-5)
Main results	16	(*a*) Give unadjusted estimates and, if applicable, confounder-adjusted estimates and their precision (eg, 95% confidence interval). Make clear which confounders were adjusted for and why they were included (Tables 3, results para 2-5)<br>(*b*) Report category boundaries when continuous variables were categorized (methods para 7)<br>(*c*) If relevant, consider translating estimates of relative risk into absolute risk for a meaningful time period (Not applicable)

Content	Item no.	Recommendation
Other analyses	17	Report other analyses done—e.g., analyses of subgroups and interactions, and sensitivity analyses
Discussion		
Key results	18	Summarize key results with reference to study objectives (discussion para 2-4)
Limitations	19	Discuss limitations of the study, taking into account sources of potential bias or imprecision. Discuss both direction and magnitude of any potential bias (discussion para 8-11)
Interpretation	20	Give a cautious overall interpretation of results considering objectives, limitations, multiplicity of analyses, results from similar studies, and other relevant evidence (discussion para 2-5)
Generalizability	21	Discuss the generalizability (external validity) of the study results (discussion para 8)
Other information		
Funding	22	Give the source of funding and the role of the funders for the present study and, if applicable, for the original study on which the present article is based (Page 38)

*Give information separately for cases and controls in case-control studies and, if applicable, for exposed and unexposed groups in cohort and cross-sectional studies.

Note: †An explanation and elaboration article (freely available on the websites of PLoS Medicine at http://www.plosmedicine.org, Annals of Internal Medicine at http://www.annals.org, and Epidemiology at http://www.epidem.com) discusses each checklist item and gives methodological background and published examples of transparent reporting. The STROBE checklist is best used in conjunction with this article. Information on the STROBE Initiative is available at www.strobe-statement.org.
